# Knowledge, Attitudes and Practices Regarding Antibiotic Use and Resistance among Veterinary Students in Bangladesh

**DOI:** 10.3390/antibiotics10030332

**Published:** 2021-03-22

**Authors:** Lorraine Chapot, Md Samun Sarker, Ruhena Begum, Delower Hossain, Rahima Akter, Md Mehedi Hasan, Zamila Bueaza Bupasha, Md Bayzid, Md Salauddin, Md Shafiullah Parvej, AHM Musleh Uddin, Fazlul Hoque, Joya Chowdhury, Md Niyamat Ullah, Md Kaisar Rahman, Nure Alam Siddiky, Guillaume Fournié, Mohammed A. Samad

**Affiliations:** 1 Pathobiology and Population Sciences, Royal Veterinary College, London NW1 0TU, UK; lchapot18@rvc.ac.uk (L.C.); gfournie@rvc.ac.uk (G.F.); 2 Antimicrobial Resistance Action Center (ARAC), Bangladesh Livestock Research Institute (BLRI), Dhaka 1341, Bangladesh; samuncvasu@gmail.com (M.S.S.); dr.ruhenabegum@gmail.com (R.B.); nasiddiky.saarc@gmail.com (N.A.S.); 3 Department of Medicine and Public Health, Sher-e-Bangla Agricultural University, Dhaka 1207, Bangladesh; delowervet@sau.edu.bd; 4 Department of Pharmacy, World University of Bangladesh, Dhaka 1205, Bangladesh; shipa.ju93@gmail.com; 5 Department of Medicine, Bangladesh Agricultural University, Mymensingh 2202, Bangladesh; hasan.vet39856@bau.edu.bd; 6 Department of Microbiology and Veterinary Public Health, Chattogram Veterinary and Animal Sciences University, Chattogram 4225, Bangladesh; bupasha1494@gmail.com; 7 Department of Pathology and Parasitology, Chattogram Veterinary and Animal Sciences University, Chattogram 4225, Bangladesh; md.bayzid18fvm@gmail.com; 8 Department of Microbiology, Hajee Mohammad Danesh Science and Technology University, Dinajpur 5200, Bangladesh; salauddin.dvm@gmail.com; 9 Gono Bishwabidyalay, Dhaka 1344, Bangladesh; drparvejbd@gmail.com (M.S.P.); joyachowdhury.cvasu@gmail.com (J.C.); 10 Department of Surgery and Theriogenology, Sylhet Agricultural University, Sylhet 3100, Bangladesh; musleh.dst@sau.ac.bd; 11 Veterinary Medicine, Bangabandhu Sheikh Mujibur Rahman Agricultural University, Gazipur 1706, Bangladesh; bsmraufazlul@gmail.com; 12 Department of Veterinary and Animal Sciences, University of Rajshahi, Rajshahi 6205, Bangladesh; niyamat8340@gmail.com; 13 EcoHealth Alliance, New York, NY 10001-2320, USA; kaisarrahman@ecohealthalliance.org

**Keywords:** antimicrobial resistance, knowledge, attitudes and practices (KAP), Bangladesh, veterinary medicine

## Abstract

The use of antibiotics in animals for both therapeutic and non-therapeutic purposes is a major driver of the emergence and spread of antimicrobial resistance (AMR). While several studies have investigated prescription and consumption patterns in humans, little attention has been paid to the veterinary sector. A cross-sectional study was conducted in 3002 veterinary students (VS) and non-medical students (NMS) from 12 universities in Bangladesh to explore their knowledge, attitudes and practices (KAP) about antibiotics and AMR using a self-administered questionnaire, and assess the influence of the veterinary curriculum. KAP regarding antibiotic use and AMR was significantly higher in veterinary than non-medical students, and in first-year than final-year students. However, gaps in knowledge and practices were highlighted, suggesting deficiencies in training. Moreover, final-year veterinary students were found to be more likely than first-year students to use antibiotics without instructions, which could indicate deficiencies in their curriculum. Although the study suggested a positive impact of the veterinary curriculum on KAP about antibiotics and AMR in Bangladesh, critical gaps remain that are likely to contribute to inadequate use in their future practice. Therefore, there is scope for improving educational programs on AMR in professional curricula.

## 1. Introduction

Over the past decades, antimicrobial resistance (AMR) has emerged as one of the greatest global public health threats, compromising our ability to control infectious diseases and undermining essential medical advances [1,2]. Currently, it is estimated that AMR is responsible every year for at least 700,000 deaths worldwide. If no further actions are taken to address this threat, the number of annual deaths is projected to reach 10 million in 2050, with nearly half of these occurring in Asia [3]. Its consequences remain largely underestimated in low- and middle-income countries (LMIC) where antibiotics are extensively used to compensate for poor sanitary conditions, lack of diagnostic tools and limited access to alternative treatments [1,4,5,6].

While any use of antimicrobials can contribute to AMR emergence, it is now widely acknowledged that excessive usage and sub-therapeutic dosage are the main drivers of resistance [1,7,8,9]. As the development of novel replacement drugs is by far outpaced by the speed of AMR emergence and spread [1,2,3], greater emphasis should be placed on promoting appropriate use of antimicrobials. Several studies have demonstrated that mis-usage was mostly attributable to a lack of awareness among practitioners and poor adherence of users to prescription guidelines [4,10,11,12,13]. Furthermore, self-medication remains a widespread practice in many developing countries. This is facilitated by the availability of drugs over-the-counter despite increasing efforts to legislate [4,11,14,15,16,17]. Health professionals can play an essential role in AMR risk mitigation by raising patients’ awareness about the consequences of improper use and non-adherence to dosage regimens. It is therefore crucial to ensure they have adequate knowledge to adopt rational prescribing practices [9,10,11,18,19]. Indeed, WHO’s 2015 Global Action Plan (GAP) emphasized the urgent necessity to make AMR a core component of health education [20].

In line with this objective, recent studies have targeted medical students to better understand the factors influencing antimicrobial use by future prescribers [12,19,21,22,23,24,25], but few have conducted similar investigations in veterinary students [6,13,26]. Their role in the implementation of preventive measures is, however, of great importance: it has been estimated that more than half of the total amount of antimicrobials used worldwide are consumed by food-producing animals [5,8,9,27]. With a growing body of evidence highlighting their role as potential reservoirs of zoonotic resistant pathogens, antimicrobial use in animal production is a major concern [2,5,6,8,27]. In particular, the extensive use of antimicrobials for non-therapeutic purposes, including metaphylaxis and growth promotion, has been identified as a strong determinant of the emergence and spread of AMR [8,9,11,28,29]. Despite an increasing number of countries introducing regulation, enforcement is often limited by a lack of commitment from stakeholders and insufficient monitoring of AMR prevalence and antimicrobial use in animals [2,8,9,30].

In Bangladesh, the government established a National Action Plan (BNAP) in May 2017 to address the issue of AMR in line with the WHO GAP objectives [20,31,32]. However, the lack of surveillance frameworks, limited resources and poor awareness among professionals and policy makers are still hindering its effective implementation [14]. Unlicensed drug shops continue to provide easy access to antibiotics without prescription for both humans and animals, while unregulated drug promotion by pharmaceutical companies exacerbates indiscriminate consumption [29,33,34]. Furthermore, important policy gaps remain regarding antimicrobial stewardship and monitoring of use in the veterinary sector [32,35]. Unlike other countries such as Thailand, the BNAP does not set clear objectives for reducing non-therapeutic use in animals. Although it mentions the inclusion of AMR and rational antimicrobial administration practices in all health curricula, there is little evidence of its implementation in veterinary schools [32]. Studies investigating knowledge, attitudes and practices (KAP) among health professionals and students in Bangladesh are scarce and have focused mostly on human health professionals [18]. In this study, we aim to assess KAP associated with antibiotic use and AMR among veterinary students (VS), and compare it with KAP among non-medical students (NMS) to assess the influence of their curriculum.

## 2. Results

We conducted a cross-sectional study from November 2019 to March 2020 to investigate KAP regarding antibiotic use and AMR among 3002 veterinary and non-veterinary students from 12 public universities in Bangladesh using a self-administered paper-based questionnaire. Participants were recruited in various faculties through opportunistic sampling. The survey questionnaire was designed with the following objectives: (1) to describe students’ KAP regarding the use of antibiotics in VS and NMS; and (2) to compare KAP between first and final year VS to assess the impact of their curriculum.

### 2.1. Demographics

Out of the 3002 returned questionnaires, 173 were rejected because they were incomplete (*n* = 149) and/or filled by non-veterinary, but medical students (*n* = 24), leaving 2829 for analysis. Table 1 summarizes the participants’ demographic characteristics. The age of participants ranged from 17 to 29 years-old, with a mean of 21.57. Most participants were male (57.69%). There was a significantly higher proportion of males and lower proportions of last year and master’s students in VS compared to NMS. Most NMS were enrolled in studies related to agriculture (32.97%) and fisheries (17.56%).

### 2.2. Knowledge about Antibiotics and AMR

Median scores and proportions of desired answers to each question related to participants’ knowledge are presented in Table 2 and Table 3. VS scored significantly higher (14 ± 4) than NMS (10 ± 5) in their first and last year of study. Both last-year VS and NMS had higher scores than first-year students, with familiarity of antibiotics and AMR increasing in last-year VS (K2,4).

The proportion of desirable answers was higher in VS compared to NMS for all questions. While a majority of students in both groups knew about antibiotics (K2), VS had a better understanding of AMR than NMS (K4,6) and could better identify antibiotics (K15). Large differences were also observed for questions K8, 9 and 10, with a much greater proportion of VS being aware that antibiotics could be used against bacteria (89.15% vs. 48.89%) but not against viruses (69.82% vs. 48.39%) compared to NMS, suggesting a greater confusion between viruses and bacteria in NMS. Only 38.97% of NMS believed that antibiotics would not speed up the recovery of common cold compared to 56.79% of VS.

Responses to questions K11 and K14 suggested discrepancies between the existing regulation on antibiotics purchases and use, and its enforcement. Despite a large majority of VS (93.49%) and NMS (88.72%) agreeing that antibiotics should be used according to a professional’s guidelines, 47.13% and 41.54% reported that antibiotics could be obtained without prescription at pharmacies. Interestingly, this proportion was significantly higher in VS. Furthermore, only 44.68% of VS and 36.55% of NMS declared that antibiotics could not be used for disease prevention. Few students were able to mention any prohibited antibiotics.

The academic curriculum was the most prevalent source of information about antibiotics among students from both groups, followed by social networks, newspaper and television (Table 4). VS relied significantly more on their curriculum and veterinary professionals while NMS relied more on television.

### 2.3. Attitudes Regarding Antibiotics

The proportions of desirable answers to questions exploring attitudes are presented in Table 5. VS answered all questions significantly better with a median score of 59 compared to 53 for NMS. Awareness of AMR (A1,2) and risky practices (A14,15) improved in both groups from first to last year of study. Final-year VS were more aware than first-year students of the importance of vaccination in disease prevention (97.92% compared to 90.04%) and how it can help reduce the use of antibiotics (93.75% and 80.07%), while there was no difference in NMS. It is noteworthy that unlike VS, a majority of NMS seemed to support the use of antibiotics as growth promoters in livestock production.

The results also showed that VS were provided more extra-curricular opportunities to learn about antibiotics and AMR (A12,13). The proportion of VS who had attended a workshop or seminar on antibiotics increased significantly from first (38.38%) to final year (61.46%) while it decreased from 20.16% to 10.78 % in NMS. 

Taking into consideration the particular role of agriculture students (AS) regarding the use of antibiotics in livestock, we conducted an additional analysis to further explore related attitudes in this group. The results, which are provided in Appendix A, showed that AS had lower KAP scores than VS and that their A and P scores did not significantly improve from first to final year despite better K scores. The proportions of desirable answers to questions A14 and A15, which explored more specifically the use of antibiotics in food animals, was significantly lower in AS compared to VS. In particular, a higher proportion of AS considered the use of antibiotics as growth promotors in livestock as important.

### 2.4. Practices Regarding Antibiotics Use

The proportions of desirable answers to questions about practices are presented in Table 6. First (median score: 7) and last year (7.5) VS had significantly higher scores than NMS (6 and 7, respectively). NMS were more likely to use antibiotics for common flu symptoms such as fever, coughing or obstructed nose (P8) and were less mindful of the expiry date (P4,5).

Some practices considered to be at risk for AMR were common among both groups. Self-medication was prevalent in similar proportions among VS and NMS (P2), and only 56.23% of VS and 52.39% of NMS completed their full course of treatment without interrupting it once they felt better (P1). Interestingly, this proportion decreased from first (60.15%) to last year VS (46.88%). Last year VS were also more likely to use antibiotics without instructions but less against common flu symptoms. Some participants (18.13%) reported keeping leftover antibiotics for future use or disposed of these inappropriately (P7).

When buying antibiotics, most respondents noticed the expiry date but NMS were more likely than VS to buy antibiotics according to the drug seller or relatives’ recommendations (P6).

### 2.5. Factors Influencing Knowledge, Attitudes and Practices

Results of the univariable and multivariable analysis are presented in Table 7. The academic curriculum and the year of study were the only factors statistically associated with all three KAP scores in multivariable analysis. Attitude scores were also found to increase with the age of respondents. Post-hoc comparison using Dunn’s test showed that first-year students had significantly lower KAP scores than last-year and master’s students (*p*-value < 0.05), while there was no significant difference between last-year and master’s students.

### 2.6. Association between Knowledge, Attitudes and Practices

Spearman’s rank correlation coefficient showed that the scores for K, A and P were significantly positively correlated (Table 8), with the association being stronger between knowledge and attitudes and weaker between knowledge and practices.

## 3. Discussion

To our knowledge, this is the first study investigating knowledge, attitudes and practices regarding antibiotics and AMR in veterinary students in Bangladesh. Our results showed that VS had better KAP compared to NMS, suggesting a positive impact of veterinary education, with KAP scores improving significantly over the curriculum. In particular, senior VS were more familiar with the concept of AMR and aware of the importance of vaccination to reduce antibiotic use in animals. They were also more aware of risky practices. In line with other studies, the academic background, age and year of study were identified as factors influencing KAP [21,25,26,36].

However, this study highlighted important gaps in knowledge about antibiotics among VS. About a third of VS did not know that antibiotics were ineffective against viruses and only 56.79% believed antibiotics could not speed up the recovery of common cold. Similar proportions have been observed among medical students in previous studies in India, China, Jordan and the United Arabs Emirates [12,19,22,23,36]. The large proportions of students who were aware that antibiotics could be obtained without prescription at pharmacies and used for prophylaxis might reflect current practices in the field, and the lack of regulatory enforcement over drug sales in Bangladesh. This confirms the need to improve educational training on antibiotics and AMR in the veterinary curriculum. Additionally, creating awareness campaigns on social media could be an interesting approach given the high proportion of students who rely on it as a source of information.

Evidence of improper practices were also found among VS. While self-medication seemed to be less prevalent than reported by other studies in medical students [21,36], almost half of VS were likely to interrupt treatment before its completion if they felt better. Most respondents disposed of left-over antibiotics inappropriately by burying or throwing them together with household waste, which increases the risk of environmental contamination [6,37]. Almost a fifth kept leftovers for future use, presumably without proper guidelines.

Additionally, although K, A and P were found to be statistically positively correlated, the translation of better knowledge and attitudes into adequate practices was not consistent. While knowledge and attitudes improved over the curriculum, self-medication and early interruption of treatment were more prevalent in senior VS compared to first-year students, suggesting inadequate training on rational antibiotic use during their studies. This supports the findings of previous investigations in medical students and professionals, which have highlighted a similar discrepancy between knowledge and practices [4,12,21,25,36]. However, they appeared to be less likely to inappropriately use antibiotics against common cold symptoms. This could indicate a greater familiarity with their indications, as suggested by several studies in medical students [18,21,25,36].

Overall, our study demonstrates the need to strengthen the veterinary curriculum in Bangladesh. Currently, nine institutions are offering veterinary degrees but the nomenclature for degrees, curricula and syllabuses vary from one another and international standards are not well established. Efforts are being made to meet the OIE-recommended core curriculum [38].

This study has several limitations. First, participants were recruited through opportunistic sampling, which might limit the representativeness of the sample. It is particularly worth noting that a significant proportion of the NMS interviewed in this survey were in agricultural faculties, and therefore, their opinion on non-human antibiotic use might not reflect that of the general population. This potential bias must be accounted for when looking, for instance, at the high proportion of NMS who supported the use of antibiotics for growth promotion or prophylaxis. The additional analysis we conducted in agriculture students showed that their A and P scores did not significantly improve from first to final year despite better knowledge, and revealed poorer attitudes regarding the use of antibiotics in livestock compared to VS. Considering the impact of their future professional practices on AMR, it would be relevant to conduct similar surveys in this group. Reporting bias can also not be ruled out, especially for questions about practices, as participants might be more inclined to give what they considered to be the “correct” answer rather than an accurate description of their behaviors. Nevertheless, a large proportion of students still reported inadequate practices. 

Additionally, while ensuring appropriate prescription practices in veterinarians is essential to mitigate the spread of AMR, their influence on consumers might be limited. Other studies have shown that farmers actually had little interactions with veterinarians and purchased drugs from unlicensed village doctors [33,34]. This corroborates our results showing that NMS relied mostly on sellers or relatives’ recommendations rather than professionals when buying antibiotics. Finally, it is important to acknowledge that the determinants of antibiotic use are multifactorial and that improving awareness alone is unlikely to produce sustainable results. Other barriers remain that might prevent the adoption of adequate practices despite proper knowledge including the lack of regulation frameworks and resources for their implementation, inadequate diagnostic tools and high prevalence of counterfeit drugs in LMIC [4,20,26,32].

## 4. Material and Methods

### 4.1. Study Design

A cross-sectional survey was carried out from November 2019 to March 2020 to investigate knowledge, attitudes and practices (KAP) regarding antibiotic use among 3002 students from 12 of the 53 public universities in Bangladesh using a paper-based self-administered questionnaire (Table S1). Universities were selected with the initial aim of including at least one university per Division and prioritize universities with both veterinary and non-veterinary faculties. The final selection included eight universities with both categories of faculties, three with only non-veterinary faculties and one with only a veterinary faculty in seven of the eight Divisions of Bangladesh. Veterinary students (VS) and students from non-medical faculties (NMS) (Agriculture, Business, Art, Mathematics or other sciences) were recruited through opportunistic sampling on a voluntary basis. A minimum sample size of 384 students was calculated using the formula $n=\frac{Z^{2}P\left(1-P\right)}{d^{2}}$ with a significance level α of 5%, precision d of 5% and expected prevalence of correct answers P of 0.5 [39]. The size was inflated to account for potential drop-outs and increase the significance of results. 

This study received ethical approval from the Ethical Committee of the Animal Health Research Division at the Bangladesh Livestock Research Institute (ARAC:15/10/2019:02). 

### 4.2. Questionnaire Design and Grading Method

A pilot survey was performed prior to the main study to assess the relevance and understandability of the questionnaire. Minor revisions were made afterwards. The final questionnaire included four sections: (1) collection of participants’ characteristics (age, gender, faculty, university); (2) 15 questions on knowledge about antibiotics and sources of information; (3) 15 questions exploring attitudes regarding antibiotics; and (4) 8 questions investigating practices regarding the use of antibiotics. Questions in the knowledge and practices sections were classified as “desirable” and “non-desirable” (including missing answers) and awarded 1 or 0 points accordingly. Likert scale questions in the attitude section were awarded 0 to 4 points from “Strongly incorrect” to “Strongly correct”. The grading method is summarized in Table S2. Qualitative questions that could not be graded (K5, P6 and P7) were not considered for scoring.

### 4.3. Data Collection

Ten enumerators were trained to collect the data. Before delivering the questionnaire, they explained the study objectives and required informed consent from all students who agreed to participate. All respondents had the opportunity to withdraw themselves from the study at any point. Individual questionnaires were anonymized and identified using the name of the faculty and a registration number. The data was managed using Microsoft Excel [40].

### 4.4. Data Analysis

Questionnaires with less than 70% questions answered and students from other medical faculties were not included in the analysis. Descriptive statistics and Fisher’s exact test were used to analyze and compare the characteristics of VS and NMS. Categorical variables were expressed as percentages, continuous variables as the mean ± standard deviation and discrete variables as the median and associated interquartile range (IQR). The proportions of answers to each categorical KAP question were compared between VS and NMS using Fisher’s exact test. The Shapiro–Wilcoxon test allowed us to reject the hypothesis of normal distribution of KAP scores (*p*-value < 0.001). Mann–Whitney U and Kruskal Wallis tests were used to identify factors associated with KAP scores in univariate analysis, and Spearman’s rho was used to explore the association between KAP scores. Multivariable analysis was carried out using a negative binomial regression model to account for overdispersion of the data. A *p*-value < 0.05 was taken as statistically significant. All analyses were performed using R [41].

## 5. Conclusions

Our study revealed critical gaps in KAP regarding antibiotic use and resistance in veterinary students that are likely to contribute to inappropriate use in the future. This indicates deficiencies in their training and confirms the need to strengthen educational programs on AMR in the veterinary curriculum. While improving awareness and understanding of AMR to promote rational use in professionals is an important component of mitigation strategies, it needs to be supported by policies through the implementation of formal frameworks and enforcement of regulation across all sectors. Therefore, educational interventions must be embedded in a multi-sectoral strategy involving policy makers, health practitioners, animal production stakeholders, pharmaceutical companies and consumers to effectively address the issue of AMR in a “one health” approach.

## Figures and Tables

**Table 1:** Characteristics of participants.

Characteristic	% (n) Total (*N* = 2829)	% (n) VS (*N* = 1428)	% (n) NMS (*N* = 1401)	*p* (Fisher’s Test)
**Gender**				<0.001
Female	42.31 (1197)	28.99 (414)	55.89 (783)	
Male	57.69 (1632)	71.01 (1014)	44.11 (618)	
**Age (mean ± SD)**	21.58 ± 1.71	21.52 ± 1.69	21.64 ± 1.73	0.079
17–20	28.35 (802)	28.78 (411)	27.91 (391)	
21–24	66.35 (1877)	66.87 (955)	65.81 (922)	
25+	5.30 (150)	4.34 (62)	6.28 (88)	
**Year of study**				<0.001
First (undergraduate)	18.35 (519)	18.98 (271)	17.70 (248)	
Intermediate years	65.68 (1858)	72.20 (1037)	58.60 (821)	
Last (undergraduate)	11.59 (328)	6.72 (96)	16.56 (232)	
Master’s	4.38 (124)	1.68 (24)	7.14 (100)	
**Faculty**				-
Agribusiness & Marketing	3.39 (96)	-	6.85 (96)	
Agricultural engineering & Technology	1.34 (38)	-	2.71 (38)	
Agriculture	11.59 (328)	-	23.41 (328)	
Fisheries	8.69 (246)	-	17.56 (246)	
Food Sciences	0.67 (19)	-	1.34 (19)	
Animal Husbandry	2.76 (78)	-	5.58 (78)	
Veterinary Medicine	50.48 (1428)	100 (1428)	-	
Biological Sciences	4.70 (133)	-	9.49 (133)	
Business Studies	1.70 (48)	-	3.43 (48)	
Engineering	2.33 (66)	-	4.71 (66)	
Mathematical and Physical Sciences	1.91 (54)	-	3.85 (54)	
Computer Science & Engineering	2.58 (73)	-	5.21 (73)	
Social Sciences	6.82 (193)	-	13.78 (193)	
Arts	1.03 (29)	-	2.07 (29)	

VS = Veterinary Students; NMS = Non-Medical Students; SD: standard deviation.

**Table 2:** Knowledge (K), attitudes (A) and practices (P) scores in veterinary and non-medical undergraduate students.

Score Median [IQR]		Whole			VS			NMS		
	Total (*N* = 2829)	VS (*N* = 1428)	NMS (*N* = 1401)	*p*	First (*N* = 271)	Last (*N* = 96)	*p*	First (*N* = 248)	Last (*N* = 232)	*p*
Knowledge	12 [6]	14 [4]	10 [5]	<0.001	13 [4]	16 [2]	<0.001	9 [5]	11 [4]	<0.001
Attitudes	56 [13]	59 [11]	53 [14]	<0.001	57 [16]	62 [8]	<0.001	51 [15]	54 [12]	0.003
Practices	7 [3]	7 [3]	6 [4]	<0.001	7 [4]	7.5 [3]	0.026	6 [5]	7 [3]	0.008

IQR = Interquartile range.

**Table 3:** Proportion of responses to questions about knowledge.

Question (Desirable Answer)	Whole				VS			NMS		
	% (n) Total (*N* = 2829)	% (n) VS (*N* = 1428)	% (n) NMS (*N* = 1401)	*p*	% (n) First (*N* = 271)	% (n) Last (*N* = 96)	*p*	% (n) First (*N* = 248)	% (n) Last (*N* = 232)	*p*
K1. Do you know “antimicrobials”? (Yes)	84.87 (2401)	95.31 (1361)	74.23 (1040)	<0.001	92.99 (252)	98.96 (95)	0.033	65.32 (162)	69.4 (161)	0.381
K2. Are you familiar with the concept of antibiotics? (Yes)	93.46 (2644)	96.01 (1371)	90.86 (1273)	<0.001	93.36 (253)	100 (96)	0.005	83.47 (207)	90.09 (209)	0.043
K3. Do you think that antibiotics are different from antimicrobials? (Yes)	73.84 (2089)	78.85 (1126)	68.74 (963)	<0.001	78.23 (212)	82.29 (79)	0.465	68.95 (171)	62.5 (145)	0.149
K4. Do you know about antibiotic resistance? (Yes)	83.74 (2369)	89.15 (1273)	78.23 (1096)	<0.001	77.12 (209)	98.96 (95)	<0.001	65.73 (163)	81.9 (190)	<0.001
K6. What is your understanding of antibiotic resistance? (correct answer)	68.33 (1933)	80.04 (1143)	56.39 (790)	<0.001	73.06 (198)	80.21 (77)	0.174	52.42 (130)	61.64 (143)	0.043
K7. Do you know any antibiotics that are prohibited to use in human/livestock/fisheries/agriculture? (Yes)	15.59 (441)	18.63 (266)	12.35 (173)	<0.001	16.97 (46)	23.96 (23)	0.184	17.74 (44)	13.36 (31)	0.31
K8. Can antibiotics be used to cure infections caused by bacteria? (Yes)	69.21 (1958)	89.15 (1273)	48.89 (685)	<0.001	91.88 (249)	91.67 (88)	1	64.52 (160)	48.28 (112)	<0.001
K9. Can antibiotics be used to cure infections caused by viruses? (No)	59.21 (1675)	69.82 (997)	48.39 (678)	<0.001	67.53 (183)	78.12 (75)	0.052	40.73 (101)	53.88 (125)	0.005
K10. Do you think the use of antibiotic will speed up the recovery of cold, cough and other diseases caused by common flu virus? (No)	47.97 (1357)	56.79 (811)	38.97 (546)	<0.001	60.89 (165)	57.29 (55)	0.547	36.29 (90)	43.1 (100)	0.136
K11. Antibiotics should obtainable without prescription at pharmacies (Yes)	44.36 (1255)	47.13 (673)	41.54 (582)	0.007	44.28 (120)	50 (48)	0.664	35.48 (88)	46.55 (108)	0.045
K12. Antibiotics need to be used according to prescription/professional (Yes)	91.13 (2578)	93.49 (1335)	88.72 (1243)	<0.00	90.41 (245)	93.75 (90)	0.402	87.9 (218)	88.79 (206)	0.778
K13. Do you think the frequency of use of antibiotics will decrease the efficacy of drug? (Yes)	83.13 (2380)	87.82 (1254)	80.37 (1126)	<0.001	77.49 (210)	93.75 (90)	<0.001	69.35 (172)	84.05 (195)	<0.001
K14. Should we use antibiotics for disease prevention? (No)	40.65 (1150)	44.68 (638)	36.55 (512)	<0.001	41.33 (112)	53.12 (51)	0.056	33.47 (83)	39.66 (92)	0.184
K15. Among the drugs listed below, indicate which ones are antibiotics (correct answer)										
Amoxicillin	69.18 (1957)	84.73 (1210)	53.31 (747)	<0.001	73.06 (198)	97.92 (94)	<0.001	48.39 (120)	68.1 (158)	<0.001
Penicillin	78.08 (2209)	89.57 (1279)	66.38 (930)	<0.001	80.81 (219)	98.96 (95)	<0.001	58.06 (144)	76.29 (177)	<0.001
Tetracycline	64.83 (1834)	83.96 (1199)	45.32 (635)	<0.001	73.8 (200)	96.88 (93)	<0.001	45.56 (113)	57.76 (134)	0.008
Metronidazole	30.82 (872)	33.82 (483)	27.77 (389)	0.001	30.63 (83)	35.42 (34)	0.445	30.65 (76)	32.33 (75)	0.695
Ivermectin	46.80 (1324)	64.99 (928)	28.27 (396)	<0.001	49.45 (134)	92.71 (89)	<0.001	24.19 (60)	38.36 (89)	0.001
Albendazole	52.39 (1482)	71.15 (1016)	33.26 (466)	<0.001	57.2 (155)	93.75 (90)	<0.001	25.81 (64)	46.55 (108)	<0.001

**Table 4:** Sources of information about antibiotics resistance.

Source of Information	% (n) Whole (*N* = 2829)	% (n) VS (*N* = 1428)	% (n) NMS (*N* = 1401)	*p*
Academic curriculum	57.83 (1636)	70.17 (1002)	45.25 (634)	< 0.001
Social network	34.57 (978)	35.43 (506)	33.69 (472)	0.343
Newspaper	25.31 (716)	26.96 (385)	23.63 (331)	0.042
Television	19.69 (557)	17.58 (251)	21.84 (306)	0.005
Veterinary doctor	18.52 (524)	30.53 (436)	6.28 (88)	< 0.001
Human doctor	15.73 (445)	16.32 (233)	15.13 (212)	0.409
Drug seller	4.49 (127)	3.99 (57)	5.00 (70)	0.205
Radio	4.35 (123)	4.55 (65)	4.14 (58)	0.645
Para vet	1.91 (54)	2.94 (42)	0.86 (12)	< 0.001
Other	4.42 (125)	3.50 (50)	5.35 (75)	0.017

**Table 5:** Proportion of responses to questions about attitudes.

Question (Desirable Answer)	Whole				VS			NMS		
	% (n) Total (*N* = 2829)	% (n) VS (*N* = 1428)	% (n) NMS (*N* = 1401)	*p*	% (n) First (*N* = 271)	% (n) Last (*N* = 96)	*p*	% (n) First (*N* = 248)	% (n) Last (*N* = 232)	*p*
A1. Antibiotic resistance is a problem in Bangladesh (Yes)	91.09 (2577)	95.03 (1357)	87.08 (1220)	<0.001	92.62 (251)	98.96 (95)	0.020	84.27 (209)	90.95 (211)	0.028
A2. At present, there is abuse on antibiotics (Yes)	89.47 (2531)	92.30 (1318)	86.58 (1213)	<0.001	91.51 (248)	97.92 (94)	0.033	83.47 (207)	90.52 (210)	0.030
A3. Antibiotic resistance can affect you and your family’s health (Yes)	78.19 (2212)	80.95 (1156)	75.37 (1056)	<0.001	81.92 (222)	94.79 (91)	0.001	76.21 (189)	70.69 (164)	0.180
A4. It is necessary to get more information about antibiotics (Yes)	92.22 (2609)	94.47 (1349)	89.94 (1260)	<0.001	91.88 (249)	97.92 (94)	0.052	89.92 (223)	89.22 (207)	0.881
A5. When a disease in an individual can’t be treated with antibiotics, how serious do you think it could be? (serious, very serious)	82.01 (2320)	84.73 (1210)	79.23 (1110)	<0.001	88.93 (241)	87.50 (84)	0.711	74.6 (185)	81.03 (188)	0.100
A6. When a disease in an animal can’t be treated with antibiotics, how serious do you think it could be? (serious, very serious)	85.12 (2408)	87.54 (1250)	82.66 (1158)	<0.001	91.88 (249)	86.46 (83)	0.155	80.65 (200)	82.33 (191)	0.641
A7. Should antibiotics be used only when prescribed by doctors? (Yes)	92.82 (2626)	95.31 (1361)	90.29 (1265)	<0.001	95.94 (260)	97.92 (94)	0.527	83.06 (206)	92.67 (215)	0.001
A8. Do you think vaccination can prevent diseases? (Yes)	87.80 (2484)	91.53 (1307)	84.01 (1177)	<0.001	90.04 (244)	97.92 (94)	0.014	83.47 (207)	84.05 (195)	0.902
A9. Do you think vaccination can help reduce the use of antibiotics? (Yes)	83.32 (2357)	87.18 (1245)	79.37 (1112)	<0.001	80.07 (217)	93.75 (90)	0.001	75.81 (188)	79.31 (184)	0.383
A10. Is it necessary to establish a course on “Rational use of antibiotics” at the university level? (Yes)	86.14 (2437)	89.29 (1275)	82.94 (1162)	<0.001	91.88 (249)	95.83 (92)	0.250	82.66 (205)	85.34 (198)	0.457
A11. Please rate your interest in learning more about antibiotics (interested, very interested)	90.00 (2546)	93.07 (1329)	86.87 (1217)	<0.001	90.77 (246)	96.88 (93)	0.071	84.68 (210)	86.64 (201)	0.603
A12. Have you ever attended any training/conference/seminar/workshop on antibiotics? (Yes)	25.87 (732)	36.27 (518)	15.27 (214)	<0.001	38.38 (104)	61.46 (59)	<0.001	20.16 (50)	10.78 (25)	0.005
A13. Have you ever attended any training/conference/seminar/workshop on antibiotic resistance? (Yes)	25.98 (735)	36.90 (527)	14.85 (208)	<0.001	42.07 (114)	57.29 (55)	0.012	22.18 (55)	11.21 (26)	0.001
A14-1. Antibiotics protect both humans and animals (livestock, fisheries) (agree, strongly agree)	79.36 (2245)	85.85 (1226)	72.73 (1019)	<0.001	85.24 (231)	91.67 (88)	0.117	65.73 (163)	80.6 (187)	<0.001
A14-2. Antibiotics abuse is the main cause of bacterial resistance (agree, strongly agree)	76.86 (2146)	85.15 (1216)	66.38 (930)	<0.001	83.76 (227)	96.88 (93)	0.001	55.24 (137)	71.12 (165)	<0.001
A14-3. When using antibiotics correctly, there is less risk of antibiotic resistance (agree, strongly agree)	76.67 (2169)	83.19 (1188)	70.02 (981)	<0.001	78.97 (214)	89.58 (86)	0.021	65.73 (163)	75.43 (175)	0.022
A14-4. It is important to use antibiotics as growth promoters in livestock production (disagree, strongly disagree)	52.17 (1476)	60.92 (870)	43.25 (606)	<0.001	49.45 (134)	66.67 (64)	0.004	37.1 (92)	48.28 (112)	0.016
A14-5. It is important to use antibiotics as growth promoters in the livestock & fisheries sector (disagree, strongly disagree)	55.00 (1556)	62.32 (890)	47.54 (666)	<0.001	53.14 (144)	71.88 (69)	0.002	43.95 (109)	54.31 (126)	0.028
A14-6. Inappropriate use or half course of antibiotics leads to antibiotics resistance (agree, strongly agree)	71.12 (2012)	78.78 (1125)	63.31 (887)	<0.001	66.05 (179)	85.42 (82)	<0.001	53.63 (133)	68.1 (158)	0.001
A15-1. Apply hygiene and biosecurity measure in livestock & fisheries activities (agree, strongly agree)	82.71 (2340)	91.11 (1301)	74.16 (1039)	<0.001	85.24 (231)	97.92 (94)	<0.001	64.92 (161)	81.03 (188)	<0.001
A15-2. Apply appropriately/fully vaccination of human/animals (agree, strongly agree)	81.55 (2307)	88.80 (1268)	74.16 (1039)	<0.001	84.13 (228)	97.92 (94)	<0.001	65.32 (162)	79.74 (185)	0.001
A15-3. Using antibiotic/antimicrobial by following guideline, description, and regulation (agree, strongly agree)	79.57 (2251)	87.11 (1244)	71.88 (1007)	<0.001	82.29 (223)	96.88 (93)	<0.001	64.11 (159)	75.43 (175)	0.007
A15-4. Farmers need to be provided drug description and treatment by veterinarian/authorities when they buy/use antibiotic (agree, strongly agree)	78.19 (2212)	86.83 (1240)	69.38 (972)	<0.001	81.92 (222)	95.83 (92)	0.001	60.08 (149)	73.28 (170)	0.003
A15-5. Ensure a sufficient/appropriate withdrawal time before selling to avoid antibiotic residue in food animal (agree, strongly agree)	75.57 (2138)	84.45 (1206)	66.52 (932)	<0.001	74.17 (201)	96.88 (93)	<0.001	58.47 (145)	71.98 (167)	0.002
A15-6. Everyone should follow full course of antibiotics (agree, strongly agree)	79.22 (2241)	87.54 (1250)	70.74 (991)	<0.001	81.92 (222)	95.83 (92)	0.001	64.11 (159)	73.28 (170)	0.039

**Table 6:** Proportion of responses to questions about practices.

Question (Desirable Answer)	Whole				VS			NMS		
	% (n) Total (*N* = 2829)	% (n) VS (*N* = 1428)	% (n) NMS (*N* = 1401)	*p*	% (n) First (*N* = 271)	% (n) Last (*N* = 96)	*p*	% (n) First (*N* = 248)	% (n) Last (*N* = 232)	*p*
P1. Do you stop the use of antibiotics as soon as you feel better? (No)	54.33 (1537)	56.23 (803)	52.39 (734)	0.042	60.15 (163)	46.88 (45)	0.031	46.37 (115)	52.16 (121)	0.235
P2. Do you use antibiotics without the doctor’s instructions? (No)	70.2 (1986)	71.15 (1016)	69.24 (970)	0.267	75.28 (204)	61.46 (59)	0.012	68.15 (169)	72.84 (169)	0.272
P3. Do you ask the doctor to prescribe antibiotics for a common cold? (No)	72.43 (2049)	73.25 (1046)	71.59 (1003)	0.333	70.85 (192)	62.5 (60)	0.159	69.35 (172)	75 (174)	0.186
P4. Do you check expired date of antibiotics before using? (Yes)	83.95 (2375)	86.76 (1239)	81.08 (1136)	<0.001	87.82 (238)	96.88 (93)	0.009	81.05 (201)	83.19 (193)	0.554
P5. If the antibiotic was expired, what would you do? (Stop using)	84.16 (2381)	87.32 (1247)	80.94 (1134)	<0.001	86.35 (234)	96.88 (93)	0.004	81.05 (201)	81.9 (190)	0.816
P6. Which factors do you prioritize when buying antibiotics?										
Expiry date	58.57 (1657)	65.62 (937)	51.39 (720)	<0.001	67.16 (182)	69.79 (67)	0.703	49.60 (123)	54.74 (127)	0.274
Trusted drug store	10.64 (301)	7.77 (111)	13.56 (190)	<0.001	5.90 (16)	11.46 (11)	0.108	14.92 (37)	13.79 (32)	0.795
Brand/trademark	16.30 (461)	15.76 (225)	16.85 (236)	0.445	15.13 (41)	9.38 (9)	0.171	12.50 (31)	19.40 (45)	0.045
Drug seller’s recommendations	8.80 (249)	6.72 (96)	10.92 (153)	<0.001	8.86 (24)	5.21 (5)	0.378	12.10 (30)	9.91 (23)	0.469
Family, friends or neighbors recommendations	5.16 (146)	3.99 (57)	6.35 (89)	0.005	2.95 (8)	3.12 (3)	1	5.65 (14)	6.47 (15)	0.848
P7. What do you do with leftover antibiotics?										
Throw in garbage	41.68 (1179)	40.76 (582)	42.61 (597)	0.322	38.01 (103)	56.25 (54)	0.003	35.89 (89)	44.83 (104)	0.051
Bury in the ground	17.32 (490)	21.43 (306)	13.13 (184)	<0.001	23.99 (65)	9.38 (9)	0.002	14.92 (37)	13.36 (31)	0.695
Burn	15.52 (439)	18.49 (264)	12.49 (175)	<0.001	15.87 (43)	16.67 (16)	0.872	12.10 (30)	13.79 (32)	0.589
Give to family/friends/neighbors	4.67 (132)	4.62 (66)	4.71 (66)	0.929	5.54 (15)	3.12 (3)	0.423	8.47 (21)	3.88 (9)	0.04
Keep for future use	18.13 (513)	19.33 (57)	16.92 (237)	0.097	25.46 (69)	15.62 (15)	0.049	15.32 (38)	20.26 (47)	0.188
Other	7.81 (221)	7.07 (101)	8.57 (120)	0.142	4.80 (13)	10.42 (10)	0.082	7.66 (19)	9.48 (22)	0.516
P8. Do you use antibiotics for the following cases? (No)										
Fever (less than 38.5 °C)	61.89 (1751)	69.61 (994)	54.03 (757)	<0.001	66.79 (181)	88.54 (85)	<0.001	51.61 (128)	56.03 (130)	0.36
Common cold	74.27 (2101)	81.37 (1162)	67.02 (939)	<0.001	78.97 (214)	92.71 (89)	0.002	60.08 (149)	74.57 (173)	0.001
Coughing up yellow/green phlegm	44.75 (1266)	46.85 (669)	42.61 (597)	0.026	51.29 (139)	62.50 (60)	0.073	37.1 (92)	40.95 (95)	0.401
Obstructed nose with headache	59.07 (1671)	64.01 (914)	54.03 (757)	<0.001	62.36 (169)	84.38 (81)	<0.001	43.15 (107)	62.93 (146)	<0.001
Coughing lasting 2 weeks or more	23.33 (660)	21.78 (311)	24.91 (349)	0.051	14.02 (38)	24.00 (24)	0.017	17.34 (43)	24.57 (57)	0.056

**Table 7:** Knowledge (K), attitudes (A) and practices (P) scores with respect to demographics.

Variable	K Scores 13 [5]	*p* (Univariable)	*est.* ± SD (*p)* (Multivariable)	A Scores 56 [13]	*p* (Univariable)	*est.* ± SD (*p)* (Multivariable)	P Scores 7 [3]	*p* (Univariable)	*est.* ± SD (*p)* (Multivariable)
Category *									
Veterinary students (N = 1428)	14 [4]	< 0.001	0.270 ± 0.020 (<0.001)	59 [11]	<0.001	0.131 ± 0.012 (<0.001)	7 [3]	<0.001	0.122 ± 0.027 (<0.001)
Non-medical students (N = 1401)	10 [5]		reference	53 [14]		-	6 [4]		-
Genre *									
Female (*N* = 1197)	12 [5]	<0.001	reference	56 [13]	0.012	-	7 [3]	0.276	-
Male (*N* = 1632)	13 [5]		0.003 ± 0.020 (0.879)	57 [13]		−0.015 ± 0.012 (0.201)	7 [3]		
Age **									
17–20 (*N* = 802)	11 [5]	<0.001	reference	54 [13]	<0.001	-	7 [3]	0.22	-
21–24 (*N* = 1877)	13 [5]		0.089 ± 0.033 (0.008)	57 [12]		0.049 ± 0.020 (0.015)	7 [3]		
25 + (*N* = 150)	14 [4]		0.059 ± 0.046 (0.196)	58.5 [10.75]		0.060 ± 0.028 (0.031)	7 [3]		
Year **									
First (*N* = 519)	11 [5]	<0.001	reference	53 [14]	0.001	-	6 [4]	0.016	-
Last (*N* = 328)	13 [5]		0.072 ± 0.035 (0.039)	57 [13]		0.020 ± 0.021 (0.351)	7 [3]		0.100 ± 0.029 (<0.001)
Masters (*N* = 124)	13 [4.25]		0.108 ± 0.044 (0.013)	58 [8.25]		0.053 ± 0.027 (0.046)	7 [3]		0.139 ± 0.040 (<0.001)

Scores are presented as median [interquartile range], Results of the multivariable analysis are presented as estimate ± standard deviation, * Mann Whitney U test, ** Kruskal Wallis test.

**Table 8:** Spearman’s correlation coefficient between knowledge, attitudes and practices.

Variable	Knowledge	Attitudes	Practices
Knowledge	-	-	-
Attitudes	0.515 (*p* < 0.001)	-	-
Practices	0.322 (*p* < 0.001)	0.422 (*p* < 0.001)	-

## Data Availability

The data presented in this study are available on request from the corresponding author. The data are not publicly available due to privacy restrictions.

## Supplementary Materials

The following are available online at https://www.mdpi.com/2079-6382/10/3/332/s1, Table S1: List of universities; Table S2: Scoring method.

## Appendix A

**Table A1:** Knowledge (K), Attitudes (A) and Practices (P) scores in veterinary and agriculture undergraduate students.

Score (Median [IQR])	Whole				VS			AS		
	Total (*n* = 2829)	VS (*n* = 1428)	AS (*n* = 805)	*p*	First (*n* = 271)	Last (*n* = 96)	*p*	First (*n* = 156)	Last (*n* = 134)	*p*
Knowledge	12 [6]	14 [4]	11 [5]	<0.001	13 [5]	16 [2]	<0.001	11.5 [4]	11 [5]	0.012
Attitudes	56 [13]	59 [11]	55 [10]	<0.001	57 [16]	62 [8]	<0.001	53 [10]	55 [10]	0.146
Practices	7 [3]	7 [3]	7 [3]	0.011	7 [4]	7.5 [3]	0.026	6 [4]	7 [3]	0.265

**Table A2:** Proportion of responses to questions about attitudes in veterinary and agriculture students.

Question about Attitudes (Desirable Answer)	Whole		
	% (n) VS (*N* = 1428)	% (n) AS (*N* = 805)	*p*
A14-1. Antibiotics protect both humans and animals (livestock, fisheries) (agree, strongly agree)	85.85 (1226)	80.99 (652)	0.003
A14-2. Antibiotics abuse is the main cause of bacterial resistance (agree, strongly agree)	85.15 (1216)	73.88 (593)	<0.001
A14-3. When using antibiotics correctly, there is less risk of antibiotic resistance (agree, strongly agree)	83.19 (1188)	79.50 (640)	0.034
A14-4. It is important to use antibiotics as growth promoters in livestock production (disagree, strongly disagree)	60.92 (870)	50.31 (405)	<0.001
A14-5. It is important to use antibiotics as growth promoters in the livestock & fisheries sector (disagree, strongly disagree)	62.32 (890)	55.40 (446)	0.001
A14-7. Inappropriate use or half course of antibiotics leads to antibiotics resistance (agree, strongly agree)	78.78 (1125)	72.30 (582)	0.001
A15-1. Apply hygiene and biosecurity measure in livestock & fisheries activities (agree, strongly agree)	91.11 (1301)	85.47 (688)	<0.001
A15-2. Apply appropriately/fully vaccination of human/animals (agree, strongly agree)	88.80 (1268)	86.09 (639)	0.069
A15-3. Using antibiotic/antimicrobial by following guideline, description, and regulation (agree, strongly agree)	87.11 (1244)	82.98 (668)	0.008
A15-4. Farmers need to be provided drug description and treatment by veterinarian/authorities when they buy/use antibiotic (agree, strongly agree)	86.83 (1240)	80.87 (651)	<0.001
A15-5. Ensure a sufficient/appropriate withdrawal time before selling to avoid antibiotic residue in food animal (agree, strongly agree)	84.45 (1206)	79.13 (637)	0.002
A15-6. Everyone should follow full course of antibiotics (agree, strongly agree)	87.54 (1250)	82.98 (668)	0.004
